# Investigation of the appropriate viscosity of fibrinogen in repairing pleural defects using ventilation and anchoring in an ex vivo pig model

**DOI:** 10.1186/s13019-024-02643-9

**Published:** 2024-03-21

**Authors:** Akihiro Fukuda, Masaki Hashimoto, Yoshitaka Takegawa, Nobuyuki Kondo, Seiki Hasegawa

**Affiliations:** 1 Department of Thoracic Surgery, School of Medicine, Hyogo Medical University, Mukogawa-cho 1-1, Nishinomiya city, 6638501 Hyogo Japan; 2 Research Department, KM Biologics Co., Ltd, Section 2, Kumamoto, Japan

**Keywords:** Fibrin glue, Polyglycolic acid felt, Malignant pleural mesothelioma, pleurectomy/decortication

## Abstract

**Objective:**

Our previous study revealed that the viscosity of fibrinogen could influence the effectiveness of ventilation and anchoring (V/A) methods for controlling air leakages. Here, we examined the association between the viscosity of fibrinogen and effectiveness using an ex vivo pig model.

**Methods:**

The fibrin glue used in this study was BOLHEAL® (KM Biologics Co., Ltd., Kumamoto, Japan). We prepared three types of fibrinogen with different viscosities (higher and lower than normal), including one without additives. Using an ex vivo pig model, a pleural defect was made, and the defect was repaired using three different viscosities of fibrinogen through the V/A method. We measured the rupture pressure at the repair site (*N* = 10) and histologically evaluated the depth of fibrin infiltration into the lung parenchyma at the repair sites.

**Results:**

The median rupture pressure was 51.5 (40–73) cmH2O in Group 1 (lower viscosity), 47.0 (47–88) cmH2O in Group 2 (no change in viscosity), and 35.5 (25–61) cmH2O in Group 3 (higher viscosity). There was no statistically significant difference between Groups 1 and 2 (*p* = 0.819), but the rupture pressure was significantly higher in Group 2 than in Group 3 (*p* = 0.0136). Histological evaluation revealed deep infiltration of fibrin into the lung parenchyma in Groups 1 and 2, but no such infiltration was observed in the higher-viscosity group.

**Conclusions:**

The results of this experiment suggested that the V/A method using fibrin glue containing low-viscosity fibrinogen was more effective in controlling air leakage due to pleural defects.

## Introduction

Postoperative air leakage is one of the most severe complications associated with thoracic surgery [1]. Persistent air leakage leads to not only prolonged hospital stays but also worsening quality of life. Therefore, it is crucial to control air leakage in thoracic surgery [2]. Lung parenchyma injuries and pleural defects are commonly repaired using fibrin glue (FG) and polyglycolic acid (PGA) sheets, and multiple methods using such materials have been reported [3–7]. We previously reported the ventilation and anchoring (V/A) method, which seemed to be as effective as other methods reported in the past [8].

Among all thoracic surgeries, pleurectomy/decortication (P/D) is the most frequent to encounter postoperative air leakage. P/D is indicated in the curative-intent surgery for pleural mesothelioma (PM). Prolonged air leakage is sometimes observed after P/D due to huge amount of pleural defect. Therefore, the rate of persistent postoperative air leakage is very high, with a P/D of 20–58% [9–14], which means that controlling air leakage is essential [15]. In our previous prospective clinical study using the V/A method for repairing lung parenchyma, the median duration of postoperative air leakage was 4 days as a favorable result, which suggested the method’s effectiveness [16]. However, we used two types of FG with different fibrinogen viscosities and found that FG with a lower viscosity had a significantly shorter duration of postoperative air leakage than that with a higher viscosity [16]. These results suggested that using fibrinogen with lower viscosity might contribute to the formation of fibrin at deeper lung parenchyma. This is a reason why fibrinogen with lower viscosity could easily infiltrate into the lung parenchyma and its subsequent crosslinking with thrombin, ultimately achieving strong fibrin formation. Therefore, this study investigated the impact of fibrinogen viscosity on the effectiveness of the V/A method in controlling air leakage using ex vivo pig models.

## Methods

### Use of animal materials

Extracted pig lungs were used in this study. We created a protocol for using commercially available pig lungs (Tokyo Shibaura Zoki Co., Ltd., Tokyo, Japan) and creating models in a lab. No application to or approval from an animal testing review board was needed, as we used lungs from dead animals.

### Fibrin glue

FG consists of two types of fluid: a fibrinogen solution, which is used as the substrate, and thrombin, which is an enzyme. In this study, we used FG, which exhibited a shorter duration of air leakage in our previous study (BOLHEAL^®^, KM Biologics Co., Ltd., Kumamoto, Japan), as the control (no change in viscosity). We prepared FG solutions with viscosities higher and lower than the control and compared them with the three groups (Table 1).

**Table 1:** Fibrinogen viscosity and additives in each group

	Group 1 (Lower viscosity)	Group 2 (Viscosity unchanged)	Group 3 (Higher viscosity)
Estimated viscosity (20 °C, mPa·S)	23.55	33.44	47.61
Additives	Arginine hydrochloride (12 mg/ml)	None	Mannitol (90 mg/ml)

Group 2 used conventional FG with no change in viscosity. Group 1 used FG with arginine hydrochloride (NACALAI TESQUE, INC.) added at a rate of 12 mg/ml to lower viscosity. Group 3 used FG with D-mannitol (FUJIFILM Wako Pure Chemical Corporation) at a rate of 90 mg/ml to increase viscosity. Since the using fibrin glue was lyophilised, both arginine hydrochloride and D-mannitol were completely dissolved in the fibrin glue solution beforehand, and the lyophilised powder of fibrinogen was dissolved using the solution. Concerning the concentration, it was calculated backwards by dividing the amount added from the adjusted amount of drug solution. The viscosity (20 °C, mPa·S) in these three groups was measured using a viscometer (HAAKE microviscosimeter, Thermo ScientificTM) and found to be 23.55, 33.44, and 47.61 for Groups 1, 2, and 3, respectively.

### Test Summary

We developed pleural defect models using extracted pig lungs and considered the effectiveness of the V/A method using PGA sheets and FG at three different viscosity levels. We created 11 models for each group, 10 of which were used to measure the rupture pressure and 1 to conduct a histopathological evaluation.

### Test Procedure

#### Preparation of visceral pleural defect models

**Fig. 1:**
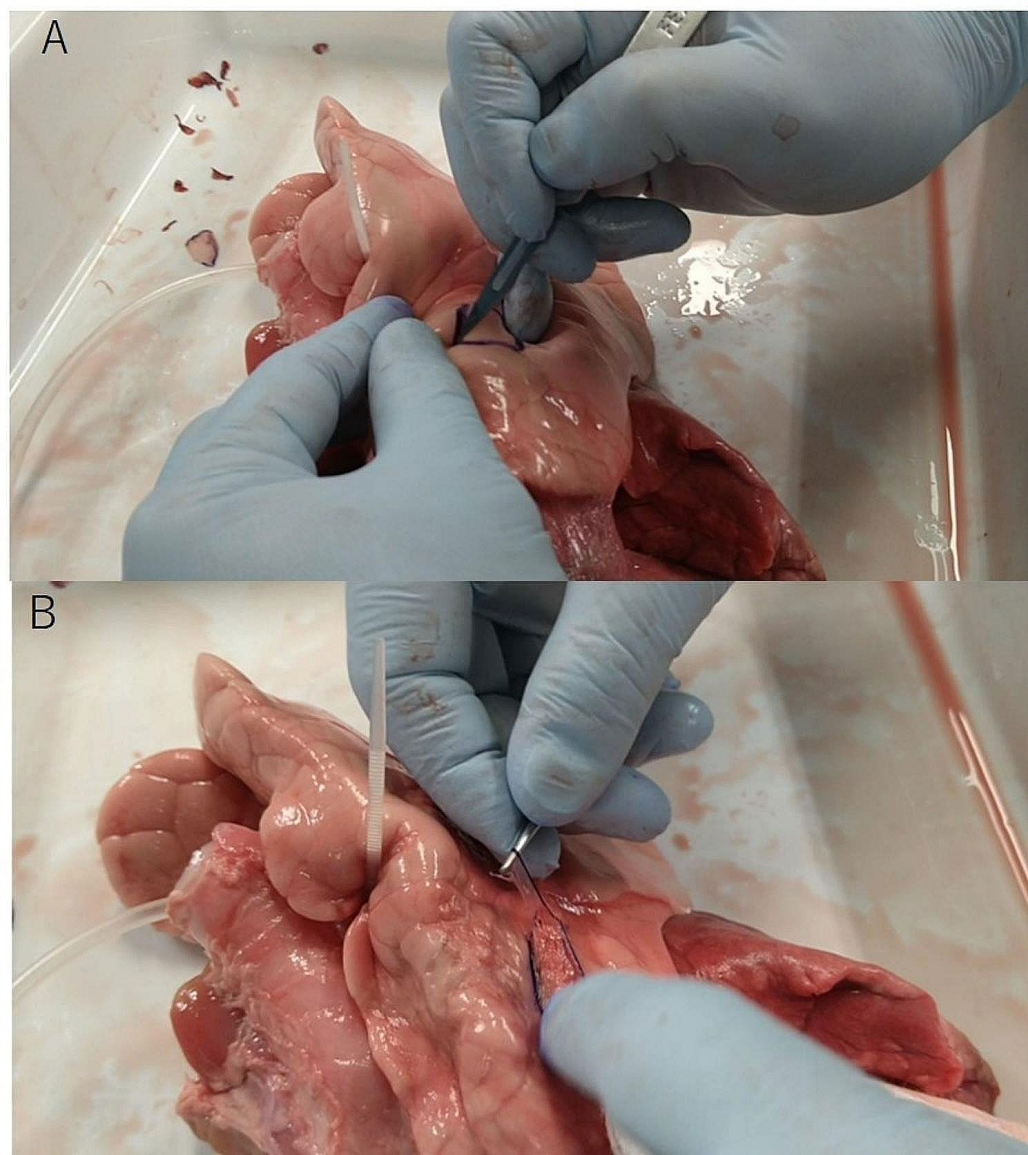
Pleural defect model. (**a**) Visceral pleura with 2 × 2 cm sharply resected (**b**) and removal of the visceral pleura to make a pleural defect model

A tube was inserted into the bronchial tube of the extracted pig lung to connect an air compressor and a pressure gauge (FUSO-8230, A-Gas Japan Co. Ltd.). While the lung was expanded, a resection area of 2 × 2 cm was marked on the surface (Fig. 1A). The visceral pleura of the marked area was then resected with a scalpel to create a pleural defect model (Fig. 1B). A single area of defect was created on one lobe.

#### Repairing pleural defects using the V/A method

As reported previously, in the V/A method, fibrinogen is applied and rubbed over the pleural defect, and the lung is ventilated a few times. A PGA sheet is then attached and sprayed with fibrinogen and thrombin [8].

**Fig. 2:**
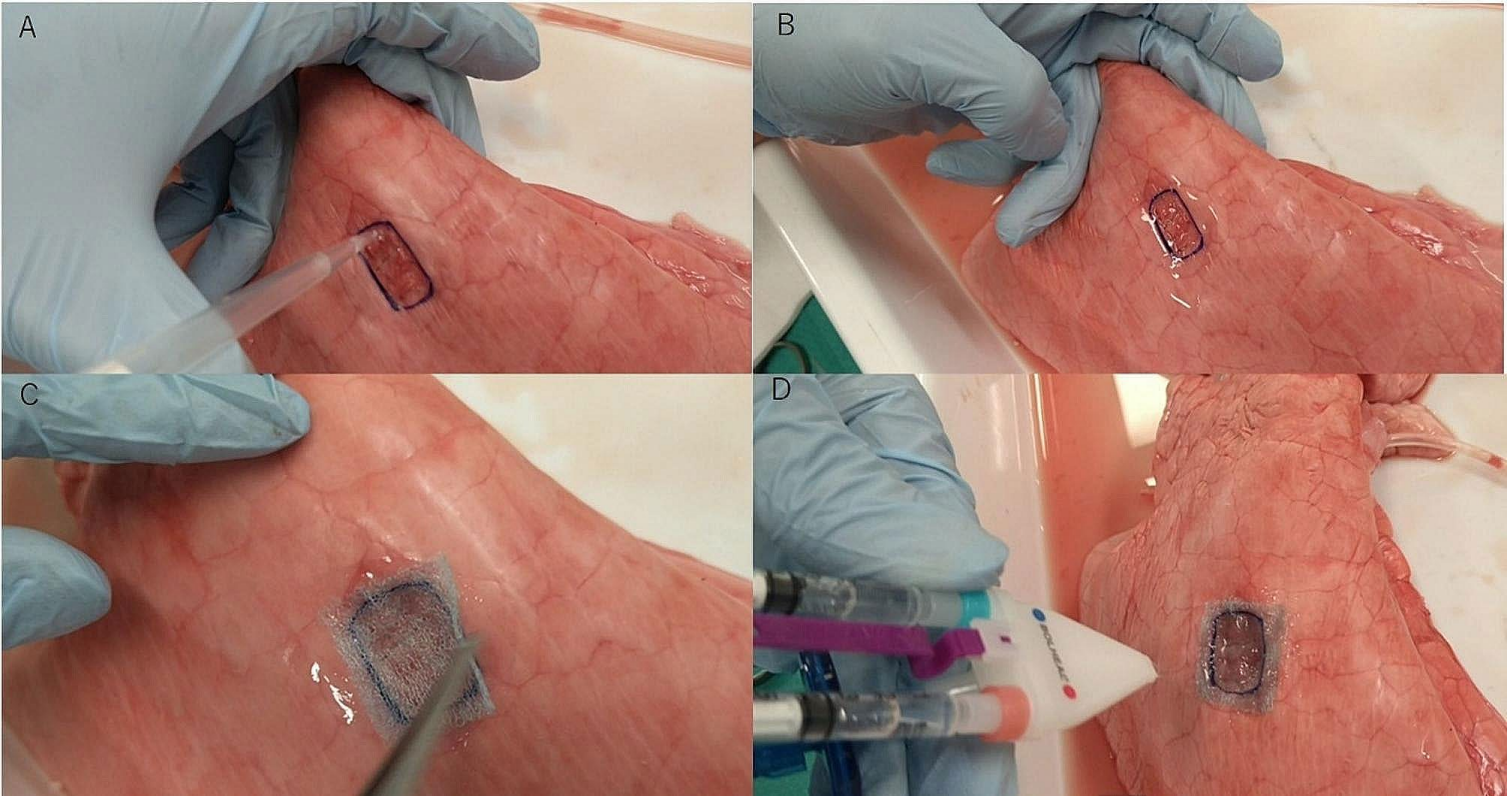
Ventilation and anchoring methods. (**a**) Fibrinogen applied to the visceral pleural defect. (**b**) Ventilated several times following the application of fibrinogen. (**c**) Pleural defect covered with a PGA sheet. (**d**) Fibrinogen and thrombin were sprayed over the site

The experiment was conducted as follows:

i) Fibrinogen solution (0.15 ml) was dropped onto each pleural defect area and rubbed over the area with the help of fingers (Fig. 2A).
ii) The lungs were ventilated for a few seconds (Fig. 2B).
iii) A PGA sheet trimmed to 3 × 3 cm was attached to the pleural defect (Fig. 2C).
iv) Fibrinogen solution (0.25 ml) and thrombin (0.4 ml) were sprayed over the pleural defect at the same time using a spray device (BOLHEAL Spray Set, Nipro Corporation) (Fig. 2D).
v) It was left to rest for 3 min.
vi) The extracted lung was ventilated by the ventilator. The ventilator supplied compressed air at 0.05 Mpa at a constant and stable rate via a flexible plastic tube with an inner diameter of approximately 2 mm and a length of 5 mm, with a three-way stopcock connection along the way. The extracted lungs dilated very slowly and there was no instantaneous increase in pressure. The pressure gauge measured the rupture pressure of the repaired pleural defect.

#### Histopathological evaluation

Following the repair of the pleural defects on the models using the V/A method (as described above), the repaired lesion was excised from the models with a scalpel to perform histological evaluation. The segments were fixed with a 10% neutral buffered formalin solution to make tissue specimens, which were then stained with hematoxylin–eosin for visual observation of fibrin infiltration into the lung parenchyma.

### Statistics

The Mann–Whitney U test was used to compare Group 2 with Groups 1 and 3. Although the preliminary experiment indicated that a minimum number of four samples was required to detect significant differences, considering the inherent variation that arises from using biological materials, such as pig lungs, we decided to conduct the experiment with *N* = 10 samples. Twa-tailed P<0.05 was considered significant and the statistical power was set as 80%. The effect size was computed based on prior examination results. All statistical analyses were conducted using EZR software (Saitama Medical Center, Jichi University, Saitama, Japan) [17].

## Results

### Rupture pressure

**Fig. 3:**
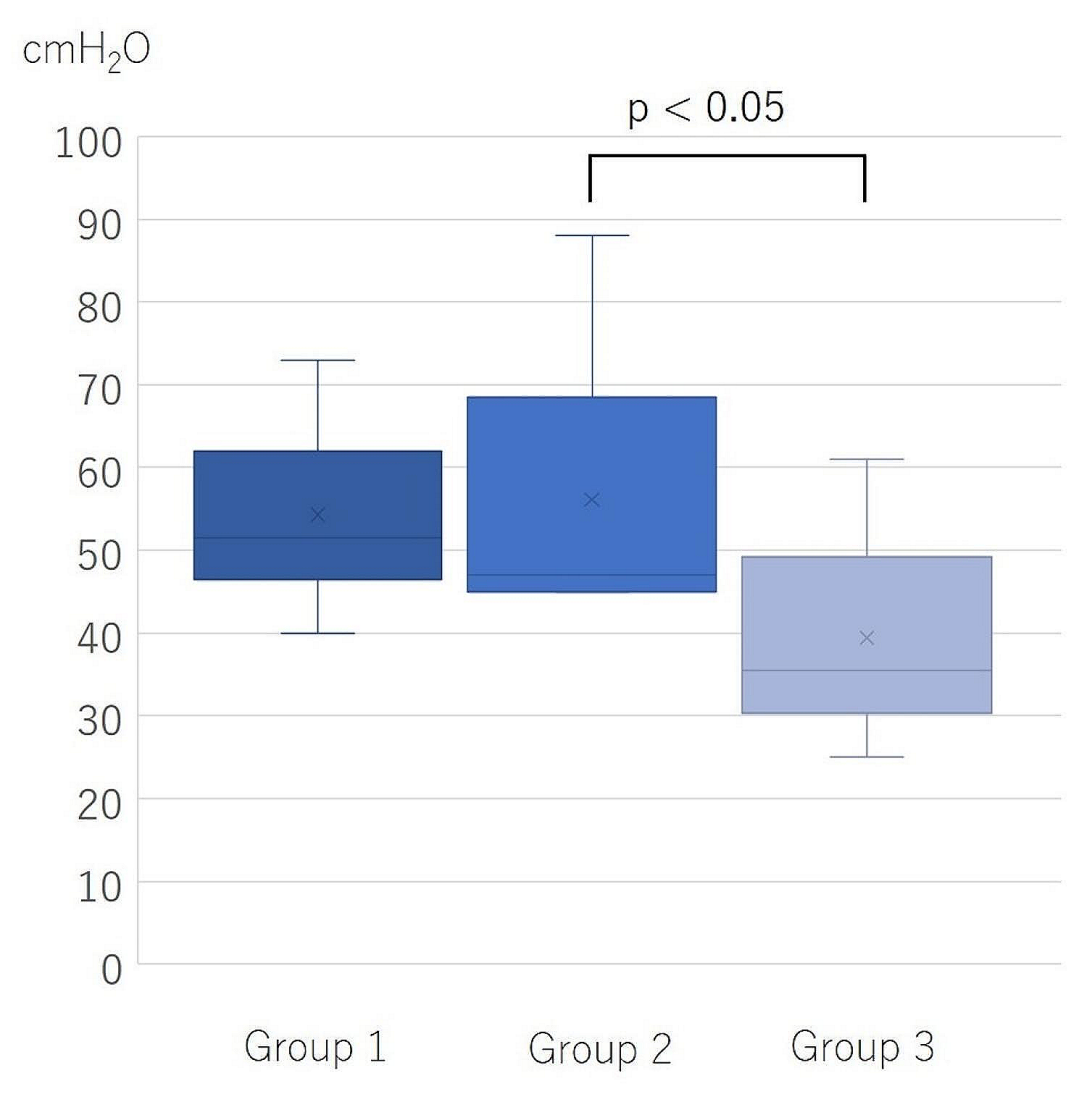
The distribution of rupture pressure. The distribution of rupture pressure in each group is shown. The comparison between Groups 2 and 3 showed that Group 2 had a significantly higher burst pressure

We measured the rupture pressure using the same method for all groups. The pressure distribution is shown in Fig. 3. The median rupture pressure (minimum–maximum) for Groups 1, 2, and 3 was 51.5 (40–73) cmH2O, 47.0 (45–88) cmH2O, and 35.5 (25–61) cmH2O, respectively. There was no statistically significant difference between Groups 1 and 2, and the rupture pressure of Group 2 was significantly higher than that of Group 3 (*p* = 0.0136).

### Histopathological evaluation

**Fig. 4:**
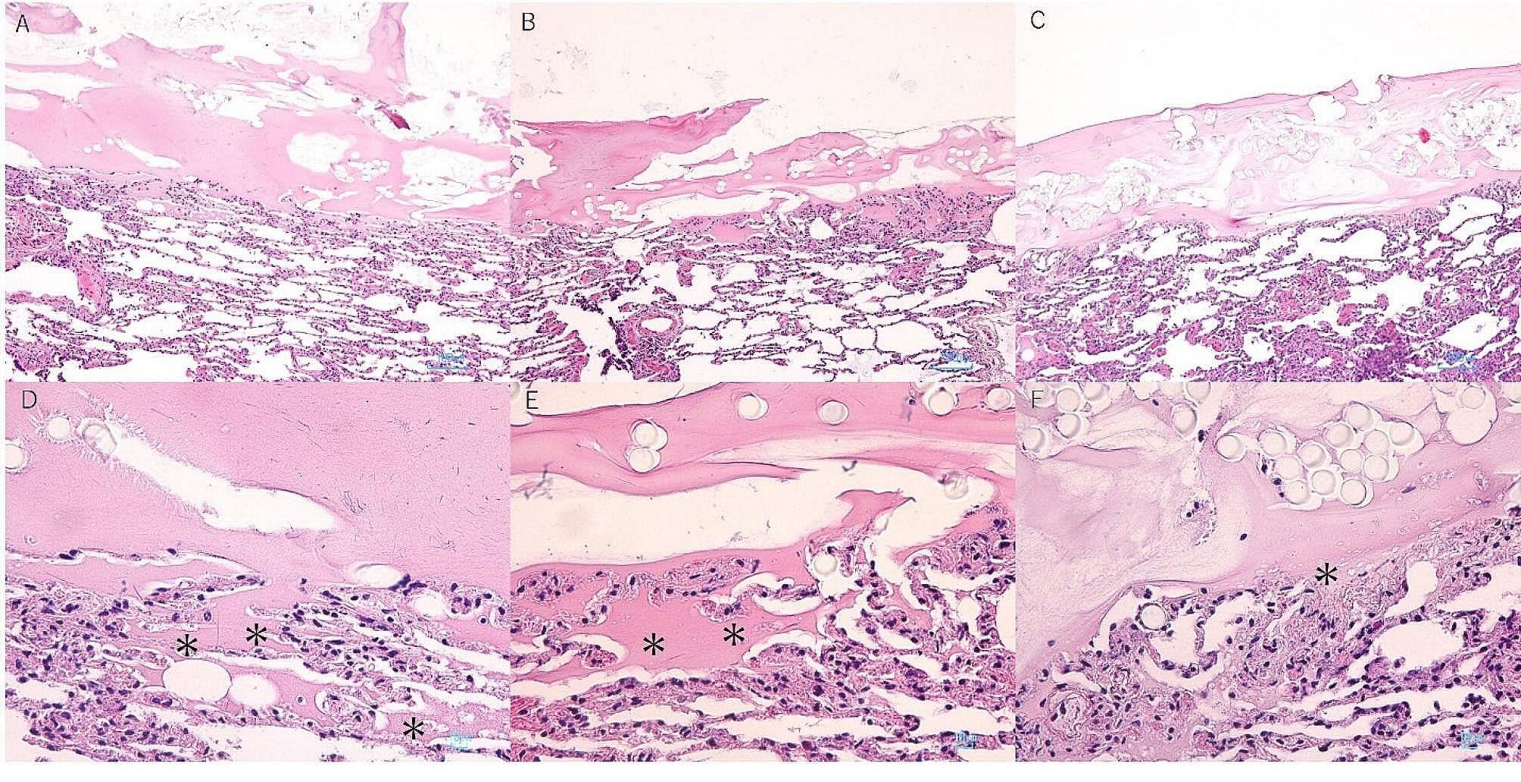
Histological findings. Histological findings at low magnification in each group (**a**: Group 1, **b**: Group 2, **c**: Group 3) showed that fibrin was present in the lung parenchymal surface (in all groups), and in Groups 1 and 2, it extended into the lung parenchyma. Histological findings at high magnification in each group (**d**: Group 1, **e**: Group 2, **f**: Group 3) revealed that in Groups 1 and 2, fibrin was widely observed in the deep regions of the lung parenchyma (marked with *), while in Group 3, only minimal fibrin was evident within the lung parenchyma

Pathological findings with high and low magnifications are shown in Fig. 4a–f. It was revealed that in Groups 1 and 2, fibrin gel infiltrated into the lung parenchyma in a wedge shape, penetrating through the surface layer. Conversely, Group 3 showed fibrin gel on the surface but hardly any fibrin gel inside the lung parenchyma.

## Discussion

To the best of our knowledge, the present study is the first to validate the influence of fibrinogen viscosity in terms of its air leakage-controlling effect. When pleural defects were repaired using the V/A method, it was observed that the rupture pressure was significantly lower in the higher-viscosity fibrinogen group than in the other groups. The histological analysis also showed that the depth of fibrin penetration into the lung parenchyma was inferior in the higher-viscosity fibrinogen group, providing additional histological support for our findings.

In our previous study, we used two types of FG (BOLHEAL^®^ and Beriplast P^®^), which showed that FG with a lower fibrinogen viscosity (BOLHEAL^®^) produced better treatment outcomes [16]. Based on these findings, we used BOLHEAL^®^ in this study and prepared three different types of FG with different levels of fibrinogen viscosity using additives.

The viscosity of Beriplast P^®^ [CSL Behring, King of Prussia, Pennsylvania, USA] used in the previous study was 42.9 [18], nearly equivalent to the viscosity adjusted to a higher level for use in this study. The results of this study revealed that fibrinogen with a lower viscosity level exhibited significantly higher rupture pressure, suggesting that the V/A method should employ fibrinogen with a lower viscosity level. This finding supports our hypothesis from the previous study that the difference in treatment outcomes was due to the viscosity of the fibrinogen. Additionally, the results of the present study support this hypothesis.

In this study, we employed the V/A method to repair pleural defects. The distinctive feature of the V/A method is the application of fibrinogen to the pleural defect site, followed by ventilation. Ventilation allows fibrinogen to be induced into the lung parenchyma, which undergoes polymerization with thrombin to form fibrin, creating wedge-shaped structures within the lung parenchyma and providing strong pressure resistance [8]. Our study revealed that the V/A method exhibits a higher pressure resistance level than other previously reported repair methods. Even in groups where high-viscosity fibrinogen was used, there was no significant difference when compared to other methods [3, 7]. These findings suggest that the V/A method is an effective approach to control air leakage and makes it a promising option for repairing pleural defects.

In this study, in addition to FG, we used a PGA sheet in repairing pleural defects. Although some repairs are conducted using only FG, previous literature has reported that combining a PGA sheet with FG provides greater pressure resistance [3, 5, 6]. In our preliminary experiments, the group using only FG did not achieve sufficient pressure resistance (data not shown). In the present study, histopathological evaluation confirmed that the PGA sheet effectively covered the pleural defect, suggesting that it plays a significant role in achieving high-pressure resistance, as observed in a previous study [8].

For surgeries where air leakage may occur from various surfaces, such as in P/D, using the V/A method with both PGA sheets and FG for lung repair seems desirable. With recent clinical trials demonstrating the noninferiority of sublobar resection compared with lobectomy for non-small cell lung cancer [19, 20], it is expected that the frequency of sublobar resections, including segmentectomy, will significantly increase. In these procedures, air leakage from the intersegmental planes is often observed, and the V/A method is considered highly useful for lung parenchymal repair during sublobar resections.

Overall, the V/A method with PGA sheets and FG seems to be a valuable approach for repairing lung parenchymal defects in various thoracic surgical procedures, including sublobar resections.

This study revealed that using fibrinogen with a lower level of viscosity is effective for repairing lung parenchyma. The ability of low-viscosity fibrinogen to easily migrate into the lung parenchyma through the pleural defect is the underlying reason, as supported by histopathological evaluation. Although fibrinogen is used in various medical specialties and surgeries other than respiratory surgery, it remains uncertain whether a low viscosity level is beneficial in all applications. Low viscosity implies that the fibrinogen is more mobile, which might not be desirable in certain situations, such as reinforcing graft anastomoses in vascular surgery or closing the dura in spinal surgery, where using fibrinogen that is less prone to migration might be preferable. In the future, we aim to determine the optimal viscosity of fibrinogen for various situations where FG is used. Customizing the viscosity of fibrinogen using a single product may have a positive impact on medical economics and patient outcomes.

Some limitations need to be addressed in this study. The study was conducted using an ex vivo extracted pig model. To truly evaluate its practical usefulness in real clinical settings, prospective clinical trials are necessary. Additionally, while we altered the viscosity of a commercially available product by adding substances, it is difficult to verify whether this method was the most suitable approach for achieving the desired viscosity.

## Conclusion

We believe that when controlling air leaks caused by pleural defects using the V/A method, using FG containing low-viscosity fibrinogen is more effective. Furthermore, histopathologically, it was demonstrated that using low-viscosity fibrinogen resulted in greater penetration of fibrin into the lung parenchyma.

## Data Availability

All data generated and analyzed during this study are included in this published article.

## Abbreviations

FG: Fibrin glue
VA: Ventilation-anchoring, P/D:pleurectomy/decortication
PGA: Polyglycolic acid

## References

[1] Rice TW, Okereke IC, Blackstone EH (2002). Persistent air-leak following pulmonary resection. Chest Surg Clin N Am.

[2] Burt BM, Shrager JB (2014). Prevention and management of postoperative air leaks. Ann Cardiothorac Surg.

[3] Sakai T, Matsutani N, Kanai E, Yamauchi Y, Uehara H, Iinuma H, Kawamura M (2018). Efficacy of a sheet combined with fibrin glue in repair of pleural defect at the early phase after lung surgery in a canine model. Gen Thorac Cardiovasc Surg.

[4] Itano H (2008). The optimal technique for combined application of fibrin sealant and bioabsorbable felt against alveolar air leakage. Eur J Cardiothorac Surg.

[5] Yano T, Haro A, Shikada Y, Okamoto T, Maruyama R, Maehara Y (2012). A unique method for repairing intraoperative pulmonary air leakage with both polyglycolic acid sheets and fibrin glue. World J Surg.

[6] Gika M, Kawamura M, Izumi Y, Kobayashi K (2007). The short-term efficacy of fibrin glue combined with absorptive sheet material in visceral pleural defect repair. Interact Cardiovasc Thorac Surg.

[7] Morikawa T, Katoh H (1999). Improved techniques of applying fibrin glue in lung surgery. Eur Surg Res Eur Chir Forsch Rech Chir Eur.

[8] Kondo N, Takegawa Y, Hashimoto M, Matsumoto S, Oka S, Hasegawa S (2020). Development of an effective method utilizing fibrin glue to repair pleural defects in an ex-vivo pig model. J Cardiothorac Surg.

[9] Nakamura A, Kondo N, Nakamichi T, Hashimoto M, Takuwa T, Matsumoto S, Kuribayashi K, Kijima T, Hasegawa S (2021). Complications and predictive factors for Air Leak > 10 days with Neoadjuvant Chemotherapy followed by Pleurectomy/Decortication for malignant pleural mesothelioma. Ann Surg Oncol.

[10] Lang-Lazdunski L, Bille A, Lal R, Cane P, McLean E, Landau D (2012). Pleurectomy/decortication is superior to extrapleural pneumonectomy in the multimodality management of patients with malignant pleural mesothelioma. J Thorac Oncol.

[11] Bölükbas S, Manegold C, Eberlein M, Bergmann T, Fisseler-Eckhoff A, Schirren J (2011). Survival after trimodality therapy for malignant pleural mesothelioma: radical pleurectomy, chemotherapy with cisplatin/pemetrexed and radiotherapy. Lung Cancer.

[12] Kostron A, Friess M, Inci I, Hillinger S, Schneiter D, Gelpke H (2017). Propensity matched comparison of extrapleural pneumonectomy and pleurectomy/decortication for mesothelioma patients. Interact Cardiovasc Thorac Surg.

[13] Nakas A, Trousse DS, Martin-Ucar AE, Waller DA (2008). Open lung-sparing surgery for malignant pleural mesothelioma: the benefits of a radical approach within multimodality therapy. Eur J Cardiothorac Surg.

[14] Hashimoto M, Yamamoto H, Endo S, Okada M, Miyata H, Hasegawa S, Chida M (2021). Japanese current status of curative-intent surgery for malignant pleural mesothelioma. Ann Thorac Surg.

[15] Hasegawa S, Kondo N, Matsumoto S, Takuwa T, Hashimoto M, Kuroda A (2019). Surgical risk and survival associated with less invasive surgery for malignant pleural mesothelioma. Semin Thorac Cardiovasc Surg.

[16] Hashimoto M, Kondo N, Nakamichi T, Nakamura A, Kuroda A, Takuwa T (2022). Control of air leakage during pleurectomy/decortication by the ventilation and anchoring method. Gen Thorac Cardiovasc Surg.

[17] Kanda Y (2013). Investigation of the freely available easy-to-use software ‘EZR’ for medical statistics. Bone Marrow Transpl.

[18] Hayashi T, Hasegawa M, Inamasu J, Adachi K, Nagahisa S, Hirose Y (2014). Experimental study on the viscosity and adhesive performance of exogenous liquid fibrin glue. Neurol Med Chir (Tokyo).

[19] Saji H, Okada M, Tsuboi M, Nakajima R, Suzuki K, Aokage K, Aoki T, Okami J, Yoshino I, Ito H, Okumura N, Yamaguchi M, Ikeda N, Wakabayashi M, Nakamura K, Fukuda H, Nakamura S, Mitsudomi T, Watanabe SI, Asamura H, West Japan Oncology Group and Japan Clinical Oncology Group (2022). Segmentectomy versus lobectomy in small-sized peripheral (JCOG0802/WJOG4607L): a multicentre, open-label, phase 3, randomised, controlled, non-inferiority trial. Lancet.

[20] Stamatis G, Leschber G, Schwarz B, Brintrup DL, Flossdorf S, Passlick B, Hecker E, Kugler C, Eichhorn M, Krbek T, Eggeling S, Hatz R, Müller MR, Hillinger S, Aigner C, Jöckel KH (2022). Survival outcomes in a prospective randomized multicenter phase III trial comparing patients undergoing anatomical segmentectomy versus standard lobectomy for non-small cell lung cancer up to 2 cm. Lung Cancer.

